# Protective effects and molecular mechanisms of tea polyphenols on cardiovascular diseases

**DOI:** 10.3389/fnut.2023.1202378

**Published:** 2023-06-28

**Authors:** Jun Guo, Kai Li, Yajun Lin, Yinghua Liu

**Affiliations:** ^1^The Key Laboratory of Geriatrics, Beijing Institute of Geriatrics, Institute of Geriatric Medicine, Chinese Academy of Medical Sciences, Beijing Hospital/National Center of Gerontology of National Health Commission, Beijing, China; ^2^General Surgery Department, The First People’s Hospital of Tai’an City, Tai’an, China; ^3^Department of Nutrition, The First Medical Center, Chinese PLA General Hospital, Beijing, China; ^4^National Clinical Research Center for Geriatric Diseases, Chinese PLA General Hospital, Beijing, China

**Keywords:** aging, cardiovascular diseases, tea polyphenols, prevention, treatment

## Abstract

Aging is the most important factor contributing to cardiovascular diseases (CVDs), and the incidence and severity of cardiovascular events tend to increase with age. Currently, CVD is the leading cause of death in the global population. In-depth analysis of the mechanisms and interventions of cardiovascular aging and related diseases is an important basis for achieving healthy aging. Tea polyphenols (TPs) are the general term for the polyhydroxy compounds contained in tea leaves, whose main components are catechins, flavonoids, flavonols, anthocyanins, phenolic acids, condensed phenolic acids and polymeric phenols. Among them, catechins are the main components of TPs. In this article, we provide a detailed review of the classification and composition of teas, as well as an overview of the causes of aging-related CVDs. Then, we focus on ten aspects of the effects of TPs, including anti-hypertension, lipid-lowering effects, anti-oxidation, anti-inflammation, anti-proliferation, anti-angiogenesis, anti-atherosclerosis, recovery of endothelial function, anti-thrombosis, myocardial protective effect, to improve CVDs and the detailed molecular mechanisms.

## 1. Introduction

With the advent of an aging society, aging-related issues are becoming a growing concern (1). Aging-related diseases have also become the most common diseases among middle-aged and elderly people (1, 2). Cardiovascular disease (CVD) is closely related to aging and is a serious threat to the lives and health of middle-aged and elderly people (3). CVD is a complex disease that involves multiple environmental and genetic factors, particularly atherosclerosis (AS), which mainly affects the large and middle arteries (3). This disease is characterized by lesions in the affected arteries starting from the intima, followed by a combination of lesions, including lipid accumulation, fibrous tissue proliferation and calcification, along with degenerative changes in the middle layers of the arteries (4). The secondary lesions of AS include lipid deposition, intimal thickening, thrombosis, inflammatory cell infiltration, subintimal inflammation, vessel wall remodeling, neovascularization, plaque rupture, intraplaque hemorrhage, plaque rupture and local thrombosis, which eventually cause narrowing or blockage of the vascular lesion, resulting in ischemic injury to the affected organs (4). AS and related diseases, such as coronary heart disease (CHD) and myocardial infarction, have become serious threats to human health and have become the leading causes of high morbidity, disability and mortality worldwide (5). Hence, there is a growing interest in exploring new ways to prevent and treat aging-related CVD.

With aging, cardiomyocytes gradually develop physiological changes such as hypertrophy, senescence, lipofuscin aggregation, fibrosis, and apoptosis, which lead to cardiac hypertrophy and heart failure (6). Vascular endothelial cells, smooth muscle cells and extracellular matrix gradually change, resulting in reduced endothelial function, a thickened intima, vascular sclerosis, increased arterial pressure, reduced number of capillaries and decreased permeability, which further cause tissue ischemia and hypoxia, oxidative stress damage and necrosis (7, 8). Eventually, this causes AS, CHD, and atherosclerotic occlusive disease (8, 9). In addition, during aging, physiological changes in glucose and lipid metabolism also occur, resulting in diabetes, hyperlipidemia, and metabolic syndrome, which in turn damage cardiovascular function and can lead to diseases such as diabetic heart disease (10, 11). Therefore, blocking these mechanisms may be a therapeutic strategy to resist aging-related CVD.

Tea is the second most widely consumed beverage after water and has been consumed for thousands of years in China (12). Tea is the dried young leaves or leaf buds of *Camelliasinenis o. Ktze.*, a plant in the *Camelliaceae* family, and is used as a drink with great nutritional, health and medicinal value (13, 14). Depending on the degree of fermentation, tea is divided into six main categories: white (not fermented), green (not fermented), yellow (slightly fermented), oolong (deeply fermented), black (deeply fermented), and dark (deeply fermented) (15, 16). Tea is rich in many biologically active components, such as polyphenols, pigments, polysaccharides, alkaloids, free amino acids and saponins (15–17). Although tea contains several chemicals, tea polyphenols (TPs) play a major role in promoting health, and green tea contains far more polyphenols than other teas (12, 18–20). A large amount of evidence confirms that TPs are effective antioxidants with anti-inflammatory, antiradiation, and antiaging properties that can prevent CVD (20, 21).

The treatment methods for CVDs are mainly divided into two categories: (1) drug conservative treatment; (2) active surgical treatment. No matter which treatment method is used, it needs to be carried out on the basis of improving lifestyle, such as a light diet, rest, physical exercise, controlling weight, blood pressure, blood sugar, blood lipids, quitting smoking, and limiting alcohol. These are important lifestyle improvement measures to reduce the risk of CVDs recurrence. Compared to these traditional treatment methods, tea is widely recognized as a healthy beverage, and multiple studies have confirmed that drinking tea regularly can reduce the risk of CVDs (22–24). This may be related to various components in tea, and TPs have the effects of lowering blood lipids, antioxidation, and inhibiting thrombosis. There have been many reports describing the role of TPs in the prevention and treatment of CVDs (25–27). These articles focus on a particular component of tea or emphasize a particular CVD. In this article, we provide a detailed review of the classification and composition of teas and an overview of the causes of aging-related CVDs; then, we focus on ten aspects of the effects of TPs to improve CVD and the detailed molecular mechanisms.

## 2. Physicochemical properties and composition of TPs

Many bioactive TPs have been identified in dry tea leaves, including flavonols, flavonoids, anthocyanins, and phenolic acids (23, 24, 28, 29). Flavanols are the main components of TPs, and flavanols are dominated by catechins and their derivatives (15, 16). According to their chemical structure, catechins can be divided into four main types: (-)-epigallocatechin-3-gallate (EGCG), (-)-epigallocatechin (EGC), (-)-epicatechin-3-gallate (ECG) and (-)-epicatechin (EC) (18). EGCG is the most physiologically active substance among catechols, accounting for approximately 50%–70%, and the physiological effects of green tea are mainly exerted by EGCG (18). Unlike green tea, oolong and black teas are fermented, and their catechins are oxidized to theaflavins (including four isomers: theaflavin, theaflavin-3-gallate, theaflavin-3′-gallate and theaflavin-3,3′-gallate) (Table 1) (18). These theophyllins exert cardiovascular protective effects, but their antioxidant activity may be lower than that of catechins (18).

**Table 1:** Tea polyphenol composition of different tea leaves.

Tea	Components	References
White tea	EC, GC, EGC, CG, ECG, GCG, EGCG	(15, 16)
Green tea	EC, GC, EGC, CG, ECG, GCG, EGCG	(15, 16, 18)
Yellow tea	EC, GC, EGC, CG, ECG, GCG, EGCG	(15, 16)
Oolong tea	Catechins, gallic acid, TF, theaflavin-3-gallate, theaflavin-3′-gallate, theaflavin-3,3′-gallate, thearubigins	(18, 30, 31)
Black tea	Catechins, gallic acid, TF, theaflavin-3-gallate, theaflavin-3′-gallate, theaflavin-3,3′-gallate, thearubigins	(18, 30, 31)
Dark tea	Catechins, gallic acid, TF, theaflavin-3-gallate, theaflavin-3′-gallate, theaflavin-3,3′-gallate, thearubigins	(18, 30, 31)

CG, catechin gallate; EC, (-)-epicatechin; ECG, (-)-epicatechin-3-gallate; EGC, (-)-epigallocatechin; EGCG, (-)-epigallocatechin-3-gallate; GC, gallocatechin; GCG, gallocatechin gallate; TF, theaflavin.

## 3. Traditional risk factors for CVD

### 3.1. Abnormal blood pressure

Hypertension is a chronic CVD (32). A study showed that for every 10 mmHg reduction in systolic blood pressure, the risk of major cardiovascular events (e.g., coronary heart disease, stroke, heart failure) is significantly reduced (33). Therefore, effective control of blood pressure can reduce the incidence of cardiovascular-related diseases, morbidity and mortality (34). In addition, prehypertension is already prevalent and accompanied by increased aortic stiffness, impaired elasticity, decreased cardiac function, and diminished insulin resistance (34). Hence, hypertension is not only a chronic form of CVD but also worsens the morbidity and mortality of major CVD (35).

### 3.2. Abnormal metabolic indices

#### 3.2.1. Abnormal glucose metabolism

Abnormal fasting plasma glucose (FPG) increases the risk of CVD (36). Abnormal glucose metabolism, especially hyperglycemia, leads to oxidative stress, microvascular damage, vascular tone and endothelial damage, as well as platelet aggregation and embolism (37, 38). In addition, hyperglycemia induces certain inflammatory factors [tumor necrosis factor-α (TNF-α), interleukin-6 (IL-6), C-reactive protein (CRP), etc.] and inflammatory reactions, all of which cause varying degrees of damage to cardiomyocytes, blood vessels, and even the heart (39).

#### 3.2.2. Dyslipidemia

Lipids are the general term for neutral fats [triacylglycerols (TG) and total cholesterol (TC)] and lipids in plasma (phospholipids, glycolipids, sterols, and steroids), which are essential for the basic metabolism of living cells (40). Among them, cholesterol [low density lipoprotein (LDL) and high density lipoprotein (HDL)] and TG are closely related to the development of atherosclerosis (AS) (40, 41). Studies have shown that for every 1 mmol/L reduction in LDL-C, the risk of CVD is reduced by 21% to 24% (41, 42).

### 3.3. Poor lifestyle habits

#### 3.3.1. Smoking

It is estimated that tobacco use causes approximately 10% of CVD worldwide (43–45). Tobacco contains approximately 4,000 chemicals, of which nicotine, carbon monoxide and other components stimulate blood pressure, lead to coronary AS, increase blood and platelet viscosity, reduce the ability to dissolve blood clots and oxygen-carrying capacity of hemoglobin, and even induce ventricular fibrillation, increasing the incidence of cardiovascular events (46, 47).

#### 3.3.2. Alcohol consumption

Many studies now indicate that small amounts of alcohol consumption can moderately reduce the risk of myocardial infarction (48, 49). However, the effects of heavy alcohol use on exacerbating CVD cannot be ignored. Both long-term heavy drinking and occasional heavy drinking can, to varying degrees, decrease HDL-C, increase plasma viscosity and fibrinogen concentration, cause platelet aggregation, impair endothelial function, increase inflammatory responses, increase heart rate, and inhibit cardiac contractile function, thereby increasing the incidence of CVD, morbidity and mortality (50, 51).

#### 3.3.3. Diet

The structure, quantity, and type of diet can also influence the occurrence of cardiovascular events (52, 53). For example, a high-salt diet can exacerbate vasoconstriction, leading to elevated blood pressure and plasma cholesterol and contributing to the development of AS (52–55). Sugar can increase blood viscosity and slow blood flow, which, combined with damage to the vascular endothelium, causes the generation of a large number of atherosclerotic plaques that block blood vessels and trigger the occurrence of acute cardiovascular events (56, 57). A high-fat diet can cause obesity or overweight, leading to metabolic disorders such as hyperlipidemia, hypertension, and other CVDs (58, 59).

#### 3.3.4. Sleep and mental factors

It is reported that in patients with insomnia, serum HDL is low, while TG level is high (60, 61). In addition, CVDs are closely related to psychological conditions such as depression, chronic psychological stress, post traumatic stress disorder (PTSD), and anxiety (62).

### 3.4. Others

Numerous epidemiological studies have shown that sex, age, and family history influence the incidence and mortality rates of CVD (63, 64). With increasing age, the onset of various metabolic diseases, and the reduction in the body’s immune system, CVD increases each year (65). Moreover, the prevalence and mortality rates are higher in men than in women, especially in premenopausal women (66). Postmenopausal women lack the protective mechanisms of a specific physiological period, and with the decrease in estrogen levels, the metabolism of the body changes, leading to an increase in the incidence of CVD (65, 67).

## 4. Molecular mechanism of the cardioprotective effect of TPs

As a natural polyphenol complex, TPs are characterized by their simple availability and wide range of biological effects (68, 69). In recent years, TPs have been shown to have good preventive and curative effects against AS, thrombosis, myocarditis, coronary artery disease, antiarrhythmia and myocardial ischemia/reperfusion (I/R) injury (70). Studies have shown that the cardioprotective effects of TPs are closely related to their antioxidant, anti-inflammatory, and blood viscosity-altering characteristics (68, 70). Here, we have reviewed the relevant literature and summarized ten mechanisms of TPs associated with protection against CVD (anti-hypertension, lipid-lowering effects, anti-oxidation, anti-inflammation, anti-proliferation, anti-angiogenesis, anti-AS, recovery of endothelial function, anti-thrombosis, myocardial protective effect),. Undoubtedly, TPs can significantly reduce the risk of CVDs by reducing the factors related to CVDs.

### 4.1. Hypotensive effects

Hypertension is a major risk factor for CVD and a common disease with a high incidence worldwide that is characterized by elevated arterial pressure (71). At present, there are many drugs that treat hypertension and can effectively lower blood pressure but have large side effects and fluctuate greatly while lowering blood pressure (72, 73). Therefore, the screening of functional food factors with antihypertensive effects is critical for the prevention and treatment of hypertension. One of the pathogeneses of hypertension is elevated levels of renin, angiotensin, and aldosterone, and so patients with hypertension will experience high renin in their bodies (74). Aqueous extracts of fermented oolong and black teas strongly inhibit renin (74). In addition, supplementation with white, black and green teas in obese mice prevented the development of hypertension (75). Further analysis revealed that this antihypertensive effect was mainly associated with increased expression of antioxidant enzymes induced by TPs such as gallic acid, xanthine and flavan-3-ol (75). In a randomized, double-blind, controlled crossover study, black tea intake increased functionally active circulating angiogenic cells compared to placebo, thereby greatly offsetting the reduction in blood flow-mediated dilation due to fat intake (76). In two epidemiological studies [ATTICA and MEDiterranean ISlands (MEDIS)], green tea is rich in high levels of catechins (e.g., EGCG) compared to black tea and, therefore, significantly reduces the likelihood of hypertension in adults aged 50 years and older (77). In addition, tannins in tea have been shown to have a hypotensive effect on rats (78). Gao et al. (52, 53) found that green tea had an antihypertensive effect on hypertension induced by a high salt diet in aged male rats, and its main mechanism of action included inhibiting the activity of the renin-angiotensin II-aldosterone system, altering the expression of sodium-potassium pumps in heart, kidney and aortic tissues and increasing the synthesis of nitric oxide in endothelial cells.

### 4.2. Lipid-lowering effects

Hyperlipidemia is an important factor that induces CVD. An increase in LDL-C and a decrease in HDL-C in serum can cause arterial endothelial cell damage, increase permeability and accelerate LDL-C deposition in the subendothelium of blood vessels (79). In recent years, a large number of studies have shown that TPs can significantly reduce serum TC, TG, and LDL-C levels and increase HDL-C levels in patients with hyperlipidemia, which can protect vascular endothelial function (79). For example, serum levels of cholesterol, LDL and TG were reduced and HDL was significantly increased in experimental rats fed a high-cholesterol diet after the administration of beverages containing theaflavin and theaflavin (80). Results from a clinical trial of tea drinking habits and HDL in Chinese adults found that in people aged 60 years or older, serum HDL concentrations decreased more slowly in tea drinkers compared to non-tea drinkers, suggesting a significant association between tea consumption and HDL-C (81). In a randomized, controlled trial, ingestion of GTC for 4 consecutive weeks significantly reduced fasting serum TG levels (82). TPs have been widely demonstrated to improve lipid metabolism abnormalities by modulating gut microbial species and functions. Ma et al. (83) found that different doses of TPs could regulate intestinal redox status and the intestinal microbiota through different patterns, thus improving the disorders of lipid metabolism induced by a high-fat diet (HFD). Wang et al. (84) found that green tea leaf powder could reshape the intestinal microbiota in the cecum of mice and increase satiety hormone secretion, there by reducing lipid metabolism disorders in mice fed a HFD. Conversely, excessive intake of TPs reduced their beneficial effects on intestinal health (83). Moreover, TPs were effective in reducing leptin in rat serum and inhibiting fatty acid uptake, thereby improving lipid and antioxidant levels (85). It is worth noting that the lipid-lowering effect of black teas (such as Liubao and Pu′er teas) is increased significantly after fermentation compared to that of the raw material, probably due to the significant increase in browning and gallic acid in the tea leaves after fermentation (86).

### 4.3. Inhibiting oxidation

Oxidative stress is present throughout the pathology of AS, and another important effect of TPs is their antioxidant properties (87). Due to the number and structure of phenolic hydroxyl groups, catecholates and theaflavins are excellent electron donors and effective free radical scavengers (87). *In vitro*, the antioxidant effects of catechols and theaflavins against human LDL oxidation were similar, and the antioxidant capacity of polyphenols was in the following order: TF3 > ECG ≥ TF2B ≥ TF2A ≥ TF1 ≥ EC > EGC (88). In addition, after drinking 600 mL of green tea daily for 4 weeks, plasma levels of oxidized LDL (ox-LDL) were reduced in smokers (89). The inhibition of ROS-producing enzymes by TPs may also enhance their antioxidant effects. Both catechols and TFs inhibit the expression of inducible NO synthase (iNOS). Another physiological source of ROS occurs during the oxidation of hypoxanthine and xanthine to uric acid (87, 90). This reaction is catalyzed by xanthine oxidase, which has now been shown to be inhibited by catechol and theaflavin. Several studies have shown that catechol induces a variety of enzymes involved in cellular antioxidant defense mechanisms (87, 90, 91). Negishi et al. (91) found that oral administration of TPs for 2 weeks induced peroxidase in the aorta in spontaneously hypertensive rats. In endothelial cells, EGCG significantly induced subtilisin oxygenase-1 through activation of AKT and Nrf2, resulting in significant protection against hydroperoxide-regulated oxidative stress (87, 90). *In vitro*, TPs ameliorated heat stress injury in cardiomyocytes by upregulating Keap1-Nrf2-ARE signaling to enhance its antioxidant capacity and inducing the expression of heat shock proteins (69). Moreover, in Wistar rats, TPs attenuated the HFD-induced increase in intima-media thickness and significantly inhibited vascular oxidative damage (92). In addition, TPs can inhibit the oxidation of lipoproteins *in vivo*. In a clinical study, urinary levels of 4-O-methylglutamic acid were significantly increased after subjects took green and black tea, suggesting that intake of TPs could inhibit LDL oxidation *in vivo* (93). Besides, in a randomized, placebo-controlled, double-blind, crossover trial, green tea extract was ingested, with EGCG and EGC as the main components. Both of them rapidly bind LDL particles and reduce the degree of oxidation of LDL, thereby reducing the risk of AS associated with oxidative stress (94).

### 4.4. Inhibiting proliferation

The proliferation and migration of vascular smooth muscle cells (VSMCs) play key roles in the formation and development of AS, postvalvular restenosis and graft vascular lesions (95). *In vivo* and *in vitro* experiments showed that catechols inhibited VSMC proliferation and migration (95). Among catechols, EGC, ECG and EGCG were significantly more effective than catechins and epicatechins in preventing proliferation (95). Kim et al. (96) found that EGCG blocked the transition of VSMCs from G1 to S phase by initiating the expression of p21/WAF1, which in turn inhibited NF-kappaB and AP-1-mediated VSMC proliferation. Additionally, the antiproliferative effects of TPs include interactions with growth factors involved in the proliferation and migration of VSMCs, such as fibroblast growth factor (bFGF) (97, 98). EGCG also significantly inhibits c-Jun nuclear translocation and AP-1 binding activity and reduces iNOS expression (99). Moreover, TPs can interact with the matrix metalloprotein (MMP) system, which contributes to the migration, proliferation, and neointima formation of VSMCs after vascular injury (100). In a rat model of carotid artery injury, catechins reduced MMP-2 activity by upregulating matrix metalloproteinase (MMP)-2 and TIMP-2, thereby inhibiting neointimal proliferation and improving vascular remodeling (100). Furthermore, in a carotid artery injury model, EGCG reduced VSMC proliferation by inhibiting extracellular signal-regulated kinase (ERK), but c-jun and p38 signaling was not affected (101). Moreover, EGCG was shown to inhibit the expression of apoptosis-related proteins and attenuate apoptosis in VSMCs induced by H_2_O_2_ (102).

### 4.5. Anti-inflammation

Acute and chronic inflammation plays a key role in the development of CVD (103, 104). TPs can modulate immune responses and have potential anti-inflammatory activity. For example, in rats fed an atherosclerotic diet, the administration of 0.2% green tea extract (Polyphenon^®^) resulted in a significant reduction in serum inflammatory markers (CRP) (103). A clinical study showed that consistent use of green tea or green tea extract significantly reduced serum amyloid alpha, which is an important CVD risk factor, in obese individuals with metabolic syndrome (105). In another randomized, double-blind trial, long-term black tea consumption reduced platelet activation and lowered plasma CRP levels in healthy men, leading to long-term cardiovascular health maintenance (106). Moreover, in female rats with chronic inflammation, supplementation with TPs suppressed the innate immune response to chronic inflammation, thereby alleviating the development of myocardial fibrosis (107). In the early stages of atherosclerosis, leukocytes adhere to vascular endothelial cells and gradually migrate to the vessel wall. EGCG significantly reduced the migration of neutrophils to the endothelial cell monolayer by inhibiting chemokine production (108). *In vitro* experiments revealed that EGCG treatment inhibited TNF-α-induced adhesion of THP-1 cells to human umbilical vein endothelial cells (109). Moreover, EGCG reduced the expression of intracellular adhesion molecule 1, which affected the adhesion and migration of peripheral blood monocytes and CD8+ T cells (110). In RAW264.7 macrophages, EGCG inhibited NF-*κ*B activation and reduced lipopolysaccharide (LPS)-induced TNFα production in a dose-dependent manner (111). In obese mice fed a HFD, TPs reduced the serum levels of TNFα, IL-1β and IL-6 by inhibiting the activation of NF-*κ*B (28). Lu′an GuaPian tea, which is a green tea, is rich in kaempferol-3-O-rutinoside (KR), which can protect against cardiovascular disease by inhibiting TLR4/MyD88/NF-*κ*B signaling and protect against myocardial injury (112). In addition, endothelial cells control vascular tone and permeability and are important for maintaining vascular homeostasis (113). Reddy et al. (113) found that EGCG reduced inflammation and decreased vasodilation by inhibiting the NF-*κ*B pathway, thereby protecting against endothelial dysfunction and delaying the onset of CVD. In addition to the NF-*κ*B signaling pathway, TPs improved the species abundance of the intestinal microbiota in the cecum, thereby improving the intestinal inflammatory response (114). Additionally, TPs could increase the expression of intestinal tight junction proteins to maintain the integrity of the intestinal barrier, thereby improving intestinal flora dysbiosis and reducing systemic inflammatory responses in obese mice (28, 115).

### 4.6. Improving the vascular endothelium function

The pathophysiological features of the cardiovascular system are characterized by a decrease in protective vasoactive substances in the endothelium, which is called endothelial dysfunction (43, 44). Numerous studies have shown that TPs improve endothelial cell function, lower blood pressure and have vasodilatory effects (116–118). For example, in obese prehypertensive women, short-term daily intake of GTE could improve endothelial function (119). Excessive accumulation of ROS is one of the important causal factors leading to endothelial cell dysfunction and hypertension (120). In bovine carotid artery endothelial cells (BCAECs), TPs could inhibit ROS production by reducing nicotinamide adenine dinucleotide phosphate (NADPH) expression, thereby alleviating angiotensin (Ang) II-induced endothelial cell hyperpermeability and possibly preventing the development of CVD (120). Moreover, in endothelial cells, TPs can bind endothelial extracellular superoxide dismutase (eEC-SOD) to inhibit LDL oxidation and thus counteract atherosclerosis (121). Endothelial nitric oxide synthase (eNOS) is a source of nitric oxide in endothelial cells and plays an important role in maintaining the function of endothelial cells (122). Caveolin-1 (Cav-1) is a negative regulator of eNOS that can affect cardiovascular function in multiple ways (123). Liu et al. (123) found that in BCAECs, TPs activated ERK1/2 and inhibited p38MAPK signaling in a dose-dependent manner, downregulating Cav-1 expression and thereby protecting endothelial cells. In addition, TPs can reduce the expression and secretion of plasminogen activator inhibitor-1 (PAI-1), a regulator that plays a key role in AS and hypertensive disease, in endothelial cells in a time-and dose-dependent manner, contributing to cardiovascular protection (123). In isolated rat mesenteric arteries, (-)-epicatechin increased NO concentrations in the vasculature and promoted vasodilation by activating iberiotoxin-sensitive K^+^ channels (116). A clinical study showed that acute black tea intake could activate NO production in endothelial cells, thereby reducing the risk of CVD (124). Kim et al. (125) found that EGCG increased LC3-II production and autophagosome formation in primary bovine aortic endothelial cells (BAECs), thereby reducing lipid accumulation and improving the development of CVD.

### 4.7. Inhibiting angiogenesis

Angiogenesis is an important pathological cause of the development of CVD (126). For instance, myocardial infarction (MI) is mainly associated with partial or complete occlusion of microvessels at the site of the lesion (126). Myocardial ischemia–reperfusion mainly refers to the production of necrotic material by ischemic cells when a patient has a myocardial infarction (127). After revascularization, blood passes through the necrotic myocardium in a short time to create reperfusion damage and increase cellular necrosis, which aggravates the symptoms of infarction and leads to malignant arrhythmias (127). To combat these conditions, restoring blood supply to the infarcted area can reduce cardiac remodeling and improve myocardial function (126). Vascular endothelial growth factor (VEGF), a homodimeric vasoactive glycoprotein, is a key regulator of angiogenesis. VEGF levels are significantly elevated in the serum of patients with different CVDs and are often associated with a poor prognosis (126). A growing number of studies have shown that TPs can protect against CVD by suppressing VEGF-mediated angiogenesis. In HUVECs, EGCG blocks the formation of the vascular endothelial growth factor receptor 2 complex, which in turn inhibits VEGF-mediated angiogenesis (128, 129). In a high-cholesterol diet male New Zealand White rabbit atherosclerosis model, green tea consumption significantly reduced VEGF expression in foam cells and smooth muscle cells, and it is hypothesized that green tea may slow the progression of atherosclerosis by reducing VEGF-induced angiogenesis (128). EGCG also inhibits angiogenesis by reducing the expression of the angiogenic factor bFGF (basic fibroblast growth factor) (130). After EGCG pretreatment, endothelial cells could induce the expression of membrane-type-1 matrix metalloproteinase (MT1-MMP), which promoted endothelial cell migration, and Cav-1, which caused tube formation, was significantly decreased, suggesting that EGCG inhibits angiogenesis (131).

### 4.8. Antiatherosclerosis

AS is the underlying cause of CVD (132). The development of AS has been associated with multiple molecular mechanisms, including endothelial dysfunction, inflammation, oxidative stress, and dysfunctional lipid metabolism (132). The protective effect of TPs on AS has been widely reported (133, 134). For example, a clinical study from Japan showed that patients who consumed >3 cups of green tea/day had a lower prevalence of coronary artery disease (CAD) than those who consumed <1 cup/day, suggesting that green tea intake may help improve coronary artery atherosclerosis in the Japanese population (135). TPs inhibit oxLDL production and thus IKB kinase (IKK)-mediated NF-*κ*B activation in a dose-dependent manner and reduce the production of the proinflammatory cytokine TNF-α (134). In a mouse model of AS, EGCG reduced proinflammatory genes and increased antioxidant protein expression in the mouse aorta, and serum C-reactive protein, monocyte chelator protein-1 and ox-LDL were significantly decreased after EGCG treatment (133). Theaflavins in tea not only reduced the concentrations of *F*(2)-isoprostane, vascular superoxide, vascular leukotriene B(4) and plasma-SP-selectin in the aorta but also enhanced eNOS activity, thereby improving NO bioavailability to alleviate the development of AS in apolipoprotein E-deficient (ApoE−/−) mice (136). Changes in the gut microbiota are also closely associated with the development of AS (137). Liao et al. (137) found that TPs promoted the proliferation of intestinal *bifidobacteria* in ApoE−/− mice, thereby reducing total cholesterol and LDL cholesterol levels and reducing HFD-induced AS plaques. In addition, TPs increased the expression of autophagic markers (such as LC3, Beclin1 and p62) in the vascular wall of mice, ameliorated lipid metabolism disorders and inhibited AS plaque formation (138).

### 4.9. Inhibiting thrombosis

Platelet activation and subsequent thromboembolism are important pathophysiological mechanisms of ischemic CVD (139). The antithrombotic effect of green tea catechins is achieved mainly through the inhibition of platelet aggregation (140). EGCG has been reported to exert its inhibitory effect on platelet viability through several mechanisms: the inhibition of collagen-mediated phospholipase (PL) Cgamma2, blockade of protein tyrosine phosphorylation, and the enhancement of Ca^2(+)^-ATPase activity, thereby reducing platelet aggregation and alleviating atherothrombosis (140). In addition, GTC did not alter anticoagulant activity but mainly altered antiplatelet activity to exert antithrombotic effects in human platelet aggregation assays induced by ADP, collagen, epinephrine, and the calcium ion polymer A23187 *in vitro* (141). EGCG has also been shown to stimulate tyrosine phosphorylation of platelet-associated proteins (e.g., Syk and SLP-76) and reduce the phosphorylation levels of focal adhesion kinases, thereby improving platelet aggregation (142). Moreover, Kang et al. (143) found that catechol modulates the reduction in intracellular calcium levels in platelets, which led to Ca^2+^-ATPase activation and the inhibition of IP3 production, thereby inhibiting fibrinogen-GPIIb/IIIb binding and reducing platelet aggregation. Inflammatory and oxidative responses caused by endothelial cell injury play equally important roles in thrombosis (144). A recent study showed that EGCG combined with warfarin significantly reduced thrombus weight in a rat model of deep vein thrombosis (144). Further *in vitro* studies showed that the combination of EGCG and warfarin protected HUVECs from oxidative stress and prevented apoptosis, and the specific mechanism involved the inhibition of HIF-1α-mediated activation of PI3K/AKT and ERK1/2 signaling (144).

### 4.10. Myocardial protective effects

Ischemia is an extremely common pathological process in myocardial lesions (145). The protective effect of TPs against myocardial injury may be due to their ability to inhibit oxidative stress associated with ischemic injury (145). For example, in a cardiac hypertrophy model in rats established by abdominal aortic constriction (AC), myocardial tissue had increased malondialdehyde (MDA) levels and decreased superoxide dismutase (SOD) activity (146). In contrast, after EGCG treatment, the MDA levels in myocardial tissue decreased, and SOD activity increased. These results suggest that EGCG ameliorates myocardial injury in rats by inhibiting oxidative stress (146). In a rat model of diabetic cardiomyopathy, TPs significantly improved myocardial function in rats, and cardiomyocyte disorders and hypertrophy were significantly improved (147). An in-depth study revealed that TPs significantly upregulated LC3-II/I and Beclin-1 expression and reduced SQSTM1/p62 expression in rat myocardial tissue (147). In addition, ingestion of TPs significantly alleviated heat stress injury in hen cardiomyocytes at 38°C, as evidenced by the downregulation of myocardial injury-related indicators (LDH, CK, CK-MB and TNF-α), and the mechanism mainly involved Keap1-Nrf2-ARE and heat shock protein (Hsp)-related heat stress responses (69). Interestingly, a recent study showed that despite the low plasma concentration of polyphenols, polyphenols were transported to the arterial intima at pH 7.4 in the form of bound lipoproteins, and polyphenol levels were significantly elevated in endothelial cells and macrophages (148). Thereafter, such high local concentrations of polyphenols protect the heart through direct antioxidant effects (148). In addition, TPs alleviate myocardial fibrosis in female rats by attenuating chronic inflammation and suppressing innate immune responses (149).

**Figure 1:**
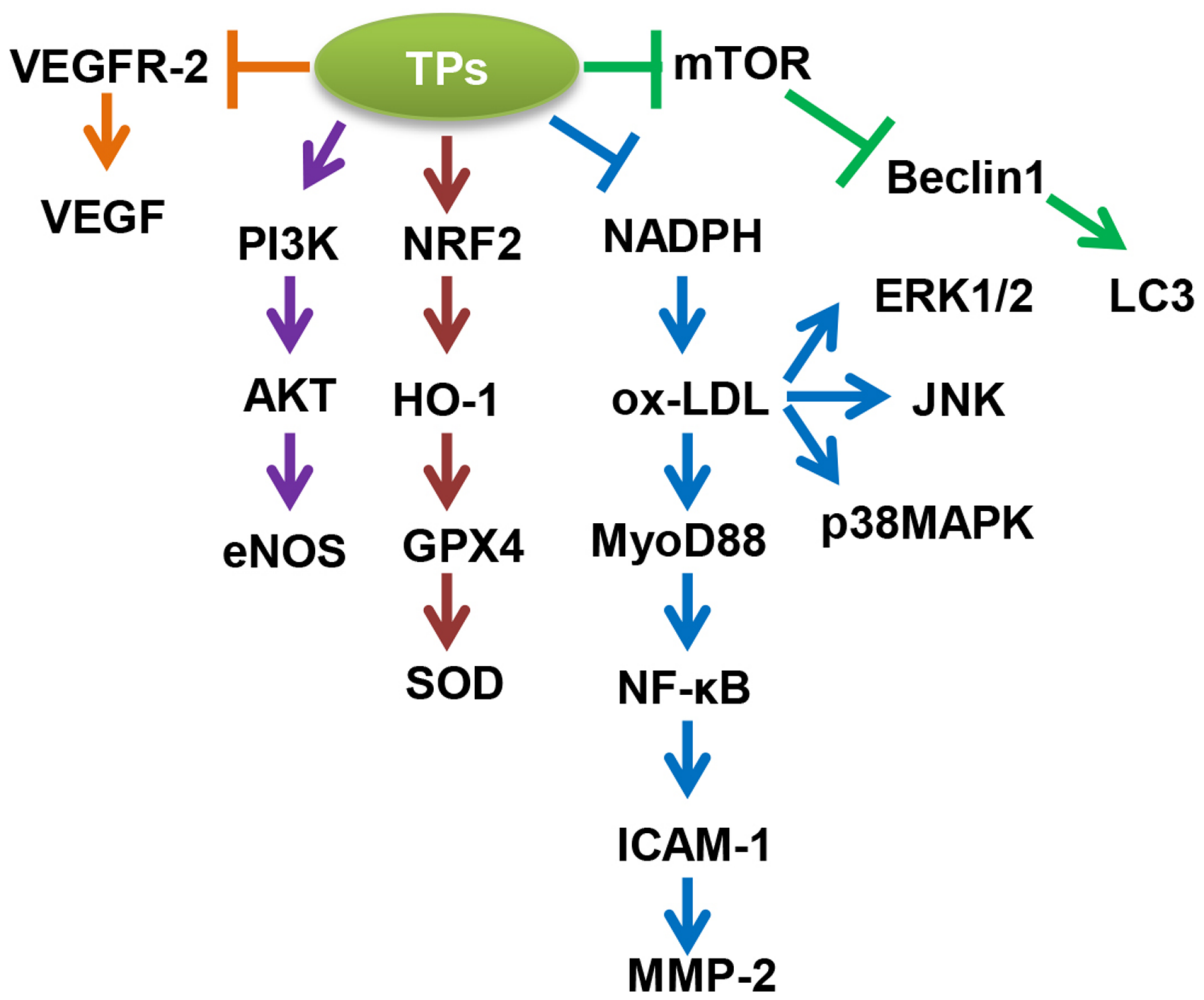
Molecular mechanism of tea polyphenols (TPs) to improve aging-related cardiovascular diseases (CVDs). AKT, AKT serine/threonine kinase 1; eNOS, nitric oxide synthase 3; ERK1/2, mitogen-activated protein kinase 1; GPX4, glutathione peroxidase 4; HO-1, heme oxygenase 1; ICAM-1, intercellular adhesion molecule 1; JNK, mitogen-activated protein kinase 8; LC3, microtubule associated protein 1 light chain 3; MMP-2, matrix metallopeptidase 2; mTOR, mechanistic target of rapamycin kinase; MyD88, MYD88 innate immune signal transduction adaptor; NADPH, 2,4-dienoyl-CoA reductase 1; NF-*κ*B, nuclear factor kappa B subunit 1; NRF2, NFE2 like bZIP transcription factor 2; ox-LDL, oxidized low density lipoprotein; p38MAPK, mitogen-activated protein kinase 14; PI3K, phosphatidylinositol-4,5-bisphosphate 3-kinase catalytic subunit beta; SOD, superoxide dismutase 1; VEGF, vascular endothelial growth factor A; VEGFR-2, kinase insert domain receptor.

Overall, TPs improve aging-related CVDs in the following five ways (Figure 1, Table 2): (1) TPs cause activation of autophagic flux; (2) TPs inhibit ox-LDL-mediated NF-*κ*B, ERK1/2, p38MAPK, and JNK-induced inflammatory responses; (3) TPs activate the NRF2-mediated antioxidant signaling pathway; 4; (4) TPs improve vascular endothelial cell function via PI3K/AKT/eNOS pathway; (5) TPs inhibit VEGF-mediated angiogenesis. By modulating these molecular mechanisms, TPs can improve aging-related CVDs.

**Table 2:** References related to molecular mechanism diagrams.

Title	References
Tea polyphenols enhanced the antioxidant capacity and induced Hsps to relieve heat stress injury	(69)
(-)-Epicatechin gallate blocks the development of atherosclerosis by regulating oxidative stress *in vivo* and *in vitro*	(87)
The inhibitory effect of (-)-epicatechin gallate on the proliferation and migration of vascular smooth muscle cells weakens and stabilizes atherosclerosis	(150)
Green tea polyphenols inhibit human vascular smooth muscle cell proliferation stimulated by native low-density lipoprotein	(151)
EGCG protects vascular endothelial cells from oxidative stress-induced damage by targeting the autophagy-dependent PI3K-AKT–mTOR pathway	(152)
(-)-Epigallocatechin-3-gallate inhibits eNOS uncoupling and alleviates high glucose-induced dysfunction and apoptosis of human umbilical vein endothelial cells by PI3K/AKT/eNOS pathway	(153)
EGCG protects against homocysteine-induced human umbilical vein endothelial cells apoptosis by modulating mitochondrial-dependent apoptotic signaling and PI3K/Akt/eNOS signaling pathways	(154)
Potent inhibition of VEGFR-2 activation by tight binding of green tea epigallocatechin gallate and apple procyanidins to VEGF: relevance to angiogenesis	(155)

## 5. Conclusion

Natural substances originating from natural food and plants are of great interest due to their low toxicity, low cost and easy availability. However, the underlying physiological mechanisms of these substances are not fully understood, especially with respect to the cardiovascular system.

**Figure 2:**
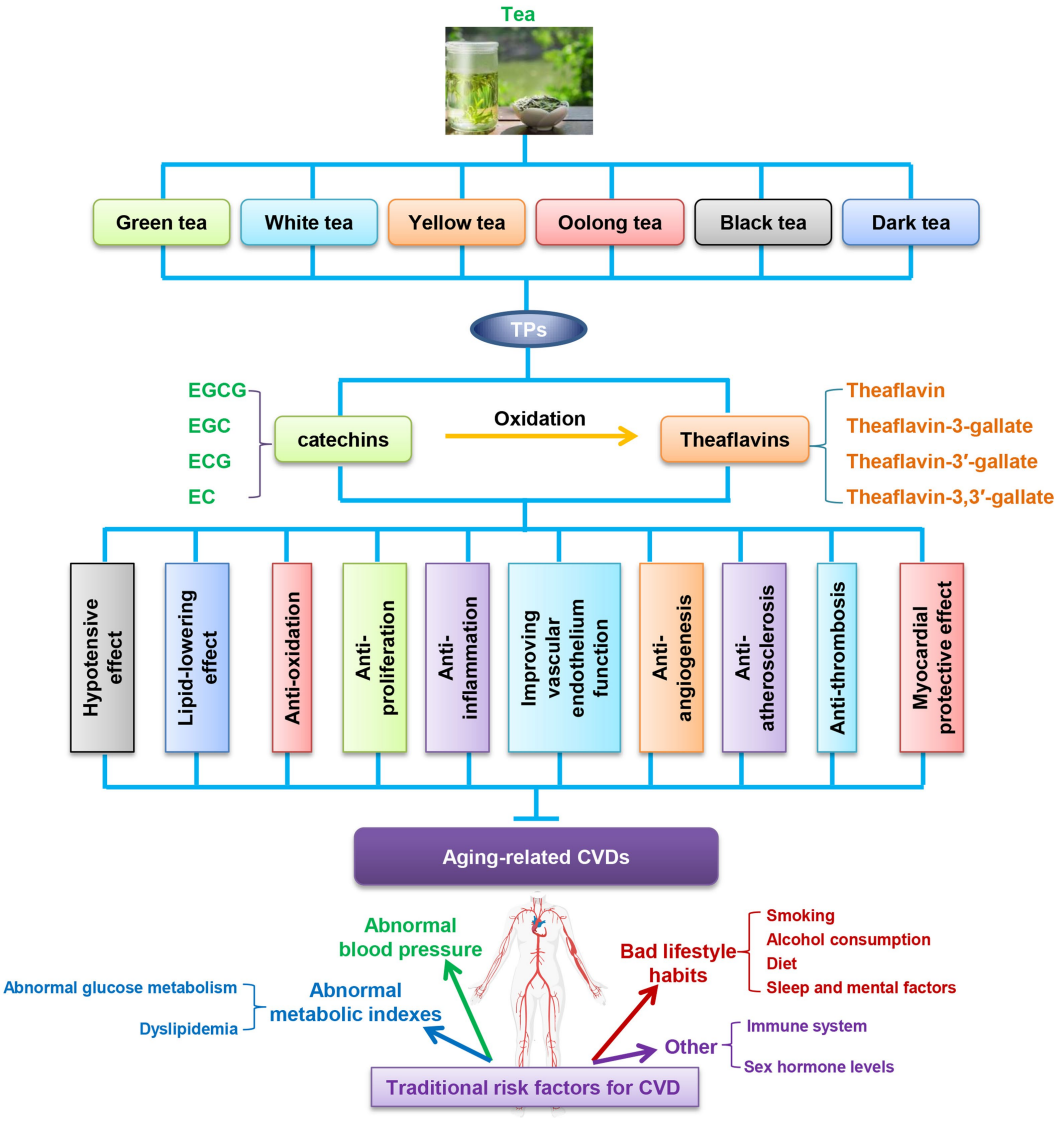
TPs improve aging related-CVDs in ten ways. CVDs, cardiovascular diseases; EC, (-)-epicatechin; ECG, (-)-epicatechin-3-gallate; EGC, (-)-epigallocatechin; EGCG, (-)-epigallocatechin-3-gallate; TPs, tea polyphenols.

The pathophysiological process of CVD is multifactorial and can be affected by tea components in several processes: anti-hypertension, lipid-lowering effects, anti-oxidation, anti-inflammation, anti-proliferation, anti-angiogenesis, anti-AS, recovery of endothelial function, anti-thrombosis, myocardial protective effect (Figure 2). However, a large number of unresolved issues exist that limit the clinical use of TPs. The debated issues are mainly related to dose, specificity, potency, feasibility and short-or long-term side effects in humans. Although naturally occurring polyphenols are generally considered pharmacologically safe, it is also important to note the presence of deleterious effects of these compounds in the body, which are largely dependent on their distribution in the body and the type of cells on which they act. In addition, the bioavailability of TPs is relatively low when administered orally, and the effective transport of TPs to target organs is an important issue (156). Moreover, some components of tea polyphenols can also interact with nutrients in the body as well as conventional drugs, which are also potential safety issues (156). To address these issues, animal experiments, large cohort studies and human intervention trials are very necessary in the future.

In conclusion, a growing body of data suggests that TPs have an important role in the prevention and treatment of CVD by interfering with multiple signal transduction pathways. However, the specific molecular roles of TPs in various cells need to be studied in great depth.

## Author contributions

JG, YaL, and YiL wrote the paper. KL collected the references. All authors contributed to the article and approved the submitted version.

## Funding

This work is supported by funds from the National Natural Science Foundation of China (82271597), the Beijing Hospital Nova Project (BJ-2020-086), and the Chinese Academy of Medical Sciences Innovation Fund for Medical Sciences (2021-I2M-1-050).

## Conflict of interest

The authors declare that the research was conducted in the absence of any commercial or financial relationships that could be construed as a potential conflict of interest.

## Publisher’s note

All claims expressed in this article are solely those of the authors and do not necessarily represent those of their affiliated organizations, or those of the publisher, the editors and the reviewers. Any product that may be evaluated in this article, or claim that may be made by its manufacturer, is not guaranteed or endorsed by the publisher.
